# A Rare Complication of Herpes Zoster: Segmental Zoster Paresis

**DOI:** 10.7759/cureus.27261

**Published:** 2022-07-25

**Authors:** Keshav R Patel, Sarah Darweesh, Darrin Lund, Kristi Vanderkolk

**Affiliations:** 1 Internal Medicine, Western Michigan University Homer Stryker School of Medicine, Kalamazoo, USA; 2 Family and Community Medicine, Western Michigan University Homer Stryker School of Medicine, Kalamazoo, USA

**Keywords:** segmental zoster paresis, herpes zoster, herpes zoster paresis, varicella zoster virus infection, stroke mimicker

## Abstract

Segmental zoster paresis (SZP) is a rare complication of herpes zoster (HZ) that results in focal weakness of the extremity in the myotome that corresponds to dermatomal involvement.We present a case of an 80-year-old female with a resolving HZ rash on her left leg and buttocks that presented with left leg weakness for two weeks. The patient’s rash preceded the left leg weakness by two weeks. The exam revealed erythematous macular and crusting lesions in the left L3/L4 distribution. The left thigh was flaccid with 1/5 knee extension strength with an absent patellar reflex. Lumbar spine magnetic resonance imaging (MRI) revealed enhancement of the left L4 roots, suggestive of inflammation or neuropathy. The patient was discharged on gabapentin, amitriptyline, and a two-week prednisone taper. In this case study, we present SZP, a rare complication that occurs in approximately 3% of HZ patients. The majority of SZP cases occur on the face or upper extremity, whereas our patient had SZP of the lower extremity. This case emphasizes the importance of maintaining a comprehensive differential diagnosis and highlights that SZP should be considered in patients who present with acute weakness in an extremity.

## Introduction

Primary varicella-zoster virus (VZV) infection, also known as chickenpox, results in a skin rash with small vesicular lesions that are in different stages and eventually scab over. After the initial infection, the virus can persist asymptomatically in the dorsal ganglia for decades. Reactivation of VZV typically occurs when there is a decrease in immunity and more commonly occurs in older adults [1-3].

Herpes zoster (HZ), also known as shingles, occurs when VZV reactivation causes a painful vesicular eruption that is usually restricted to a single unilateral dermatome [1]. The incidence of HZ is approximately 4 to 4.5 per 1000 person-years [2,3]. HZ can lead to numerous complications, including postherpetic neuralgia, encephalitis, aseptic meningitis, and segmental zoster paresis (SZP). SZP is a rare complication of HZ that results in focal weakness of the extremity in the myotome that corresponds to dermatomal involvement [3-5]. SZP affects approximately 0.5% to 5% of patients with HZ [3,4].

The pathogenesis of SZP is unclear, however, it may be due to the spread of the virus from the dorsal root ganglia to the anterior horn [4]. Most cases of SZP occur on the face (~50%), and the second most common location is the upper extremity [4]. In this case study, we present a unique case of SZP in the lower extremity.

## Case presentation

An 80-year-old female with a past medical history of hypertension, B-cell lymphoma (currently in remission), and brain aneurysm status post clipping presented to the hospital with progressive pain, numbness, and weakness of the left lower extremity for the past two weeks. The weakness was severe enough that the patient now required a walker for ambulation. A few weeks ago, the patient had been diagnosed with HZ of the left lower extremity prior to the onset of her weakness and had completed treatment with valacyclovir as an outpatient. She denied any associated constitutional symptoms at the time of presentation.

The patient’s vitals were stable on presentation. A physical exam revealed erythematous macular and crusting lesions in the anterior left L3/L4 distribution. The left thigh was flaccid with 1/5 strength on knee extension strength and an absent patellar reflex.

**Figure 1:**
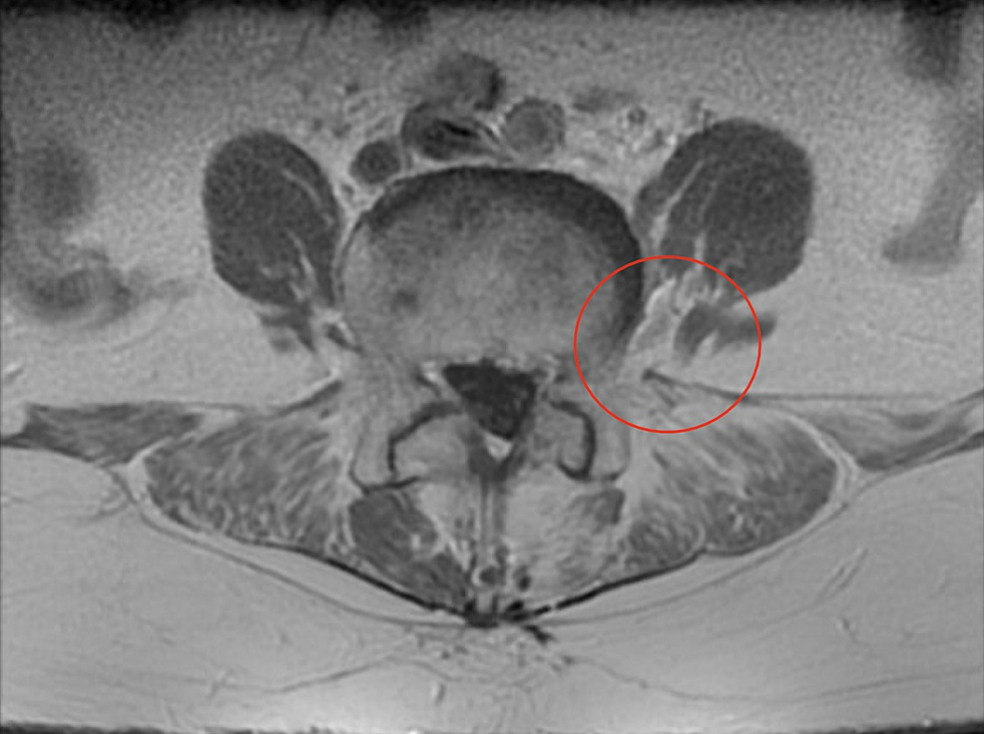
Axial magnetic resonance imaging of the lumbar spine Axial spine MRI demonstrated enhancement of the left L4 nerve roots (as indicated by the red circle) suggestive of inflammation or neuropathy. There was no evidence of masses or impingement on imaging.

Diagnostic laboratory evaluation, including a complete blood count, complete metabolic panel, and creatine kinase, was unremarkable. Magnetic resonance imaging (MRI) of the spine with and without contrast revealed enhancement of the left L4 nerve roots suggestive of inflammation or neuropathy with no evidence of masses or impingement (Figure 1).

The patient was diagnosed with SZP. As she had already completed a course of valacyclovir and did not have any active zoster lesions, she was not retreated with antiviral therapy. Her pain improved with gabapentin, nortriptyline, capsaicin, and a lidocaine patch. A prednisone taper was started at 60 mg every other day to relieve the inflammation and pain. She was discharged to a skilled nursing facility for rehabilitation of her ongoing weakness.

## Discussion

Our patient had SZP, a rare complication of HZ that results in focal weakness of the extremity in the myotome that corresponds to dermatomal involvement [3-5]. SZP occurs in approximately 3% of patients with HZ [5,6]. While most cases of SZP occur on the face (~50%) followed by the upper extremity [4], our patient presented with SZP affecting the lower extremity. A limited literature search confined to PubMed found just one article describing SZP of the lower extremity, emphasizing the uniqueness of this case.

Patients with SZP typically have MRI findings that include nerve enlargement and T2 hyperintensity consistent with the clinical symptoms [3]. Electromyography (EMG) in SZP patients may reveal low amplitude compound muscle action potentials and sensory nerve action potentials, which suggests motor and sensory axonopathy [3]. Our patient’s MRI was consistent with these typical findings, with noted hyperintensity of the left L4 nerve roots, corresponding with her myotomal involvement. Our patient did not, however, have any noted nerve enlargement on MRI. Moreover, our patient did have sensory loss that accompanied her pain and weakness, consistent with noted EMG findings in other patients. EMG was not pursued in this case, however, as history, symptoms, and imaging were all suggestive of SZP.

Of note, our patient had not previously received the Zoster vaccine, a two-dose recombinant vaccine that is extremely effective in preventing HZ and its complications [7]. Completion of both doses is >90% effective for preventing HZ and postherpetic neuralgia [7]. Previous vaccination likely would have prevented this patient’s HZ outbreak, sequelae, and subsequent hospitalization and highlights the need for continued vigilance in assuring appropriate vaccination for all patients for the prevention of morbidity and mortality. Of note, while our patient was 80 years old, there is no maximum age for receipt of the Zoster vaccine.

Finally, this case emphasizes the importance of maintaining a comprehensive differential diagnosis. SZP is a rare complication of HZ that can mimic numerous other conditions depending on the location of the initial rash. While most cases of SZP may mimic a stroke, infection, or autoimmune process, SZP can also mimic many other conditions [8]. This case highlights that SZP should be considered in patients who present with acute weakness in the extremities.

## Conclusions

SZP is a rare complication of HZ that causes focal weakness in the myotome that corresponds to the dermatome of the rash. SZP usually occurs on the face or the upper extremity; however, SZP may occur in the lower extremity as well. SZP may mimic stroke, infection, or autoimmune pathology. This case highlights that SZP should be considered in patients who present with acute weakness in the extremities, especially in patients with a recent rash. This case also emphasizes the importance of vaccination in elderly patients since previous vaccination likely would have prevented this patient's HZ outbreak, sequelae, and subsequent hospitalization.
